# Environmental surveillance for COVID-19 using SARS-CoV-2 RNA concentration in wastewater – a study in District East, Karachi, Pakistan

**DOI:** 10.1016/j.lansea.2023.100299

**Published:** 2023-10-23

**Authors:** Nadia Ansari, Furqan Kabir, Waqasuddin Khan, Farah Khalid, Amyn Abdul Malik, Joshua L. Warren, Usma Mehmood, Abdul Momin Kazi, Inci Yildirim, Windy Tanner, Hussain Kalimuddin, Samiah Kanwar, Fatima Aziz, Arslan Memon, Muhammad Masroor Alam, Aamer Ikram, John Scott Meschke, Fyezah Jehan, Saad B. Omer, Muhammad Imran Nisar

**Affiliations:** a Faculty of Health Sciences, Department of Paediatrics and Child Health, Medical College, The Aga Khan University, Stadium Road, Karachi 74800, Pakistan; b CITRIC Centre for Bioinformatics and Computational Biology, Department of Paediatrics and Child Health, Faculty of Health Sciences, Medical College, The Aga Khan University, Stadium Road, Karachi 74800, Pakistan; c Yale Institute for Global Health, Yale University, New Haven, CT, USA; d Section of Infectious Diseases and Global Health, Department of Paediatrics, Yale School of Medicine, Yale University, New Haven, CT, USA; e Yale School of Public Health, Yale University, New Haven, CT, USA; f District Health Office (East), Karachi, Pakistan; g World Health Organization: Islamabad, Chak Shahzad, Islamabad, Pakistan; h National Institutes of Health, Chak Shahzad, Islamabad, Pakistan; i University of Washington, Seattle, WA, USA; j Yale School of Nursing, Orange, CT, USA

**Keywords:** SARS-CoV-2 environmental surveillance, Karachi, Pakistan, SARS-CoV-2 genomic sequencing, SARS-CoV-2 variants, BMFS, Grab method, SARS-CoV-2 sewage surveillance, Wastewater-based epidemiology

## Abstract

**Background:**

Wastewater-based surveillance is used to track the temporal patterns of the SARS-CoV-2 virus in communities. Viral RNA particle detection in wastewater samples can indicate an outbreak within a catchment area. We describe the feasibility of using a sewage network to monitor SARS-CoV-2 trend and use of genomic sequencing to describe the viral variant abundance in an urban district in Karachi, Pakistan. This was among the first studies from Pakistan to demonstrate the surveillance for SARS-CoV-2 from a semi-formal sewage system.

**Methods:**

Four sites draining into the Lyari River in District East, Karachi, were identified and included in the current study. Raw sewage samples were collected early morning twice weekly from each site between June 10, 2021 and January 17, 2022, using Bag Mediated Filtration System (BMFS). Secondary concentration of filtered samples was achieved by ultracentrifugation and skim milk flocculation. SARS-CoV-2 RNA concentrations in the samples were estimated using PCR (Qiagen ProMega kits for N1 & N2 genes). A distributed-lag negative binomial regression model within a hierarchical Bayesian framework was used to describe the relationship between wastewater RNA concentration and COVID-19 cases from the catchment area. Genomic sequencing was performed using Illumina iSeq100.

**Findings:**

Among the 151 raw sewage samples included in the study, 123 samples (81.5%) tested positive for N1 or N2 genes. The average SARS-CoV-2 RNA concentrations in the sewage samples at each lag (1–14 days prior) were associated with the cases reported for the respective days, with a peak association observed on lag day 10 (RR: 1.15; 95% Credible Interval: 1.10–1.21). Genomic sequencing showed that the delta variant dominated till September 2022, while the omicron variant was identified in November 2022.

**Interpretation:**

Wastewater-based surveillance, together with genomic sequencing provides valuable information for monitoring the community temporal trend of SARS-CoV-2.

**Funding:**

10.13039/100005624PATH, 10.13039/100000865Bill & Melinda Gates Foundation, and 10.13039/501100024399Global Innovation Fund.

**Research in context**

**Evidence before this study**

Wastewater surveillance help in tracking diseases of public health importance. The surveillance is possible when a pathogen or its particles, such as genetic material, passes through the gastrointestinal system of humans and is detectable in the wastewater. Previous clinical studies showed the presence of SARS-CoV-2 RNA in the faeces of infected individuals. A proof-of-concept study undertaken by Ahmed and colleagues in Australia utilised the influenza surveillance infrastructure. As noted by Subissi and colleagues in their systematic review, most initial studies were carried out in high-income and middle-income countries. Wastewater surveillance could signal the outbreak of COVID-19 in a community, underpinning its potential as a disease early warning system (EWS). Genomic sequencing of samples can identify the circulating variants in a community and even detect newer variants ahead of clinical specimens. Wastewater surveillance is now being integrated as part of national SARS-CoV-2 surveillance in Australia, the UK, European Union states such as the Netherlands, Denmark, and the United States. A growing number of low-income and-middle income countries (LMICs), including Thailand, Bangladesh, India, Nepal, Ghana, and Malawi, have turned to wastewater surveillance as an EWS and for identifying the variant abundance of SARS-CoV-2 through next-generation genomic sequencing. LMICs tend to be densely populated, do not have active disease surveillance systems because of high costs and weak healthcare systems. Although wastewater surveillance has been shown to be cost-effective, limitations of infrastructure, technical and laboratory expertise, and supply constraints hinder its widespread use and implementation.

**Added value of this study**

The current study is among the first of its kind from Pakistan. It addresses some of the limitations using feasible alternative methods such as using a high-resolution digital elevation model (DEM) or ‘blue-line’ mapping for identification of sampling sites within an informal sewage system; direct sampling from main sewage stream by ‘grab method’ in the absence of functional treatment plants; on-site filtration for reduction of spills and contamination; and enhancing the capacity of in-house genomic sequencing of sewage samples. The correlation of viral RNA quantity with incident cases in the catchment area using a negative log distributed regression model showed that sewage samples could reliably predict a rise in cases with a lead time of 1–14 days.

**Implications of all the available evidence**

This study shows that wastewater sampling effectively predicts an outbreak of COVID-19 in areas with low active case surveillance or poor access to care, even without a well-mapped sewage system. As the virus continues to evolve, genomic sequencing provides additional valuable information on circulating variants.

## Introduction

Wastewater-based surveillance for SARS-CoV-2 has been used increasingly in different parts of the world for monitoring temporal trends in number of COVID-19 cases, hospitalisations and circulating variants.^1^, ^2^, ^3^, ^4^, ^5^, ^6^ Several high-income countries (HICs), with well laid out sewage networks and sewage treatment plants, have scaled up these efforts at national level. SARS-CoV-2 virus is shed in the faeces of both symptomatic and asymptomatic cases, from the time of infection to about a month post-infection.^7^^,^^8^ This means that virus can be detected in the faeces 3–7 days prior to clinical appearance of illness (in symptomatic cases).^8^ Therefore, wastewater surveillance can be used as an early disease warning system to predict number of COVID-19 cases in a defined catchment area.^4^^,^^8^ Although several low-income and-middle income countries (LMICs) have stepped up surveillance efforts,^6^^,^^9^, ^10^, ^11^, ^12^ the utility of this system in settings with a mix of formal and informal sewage network and non-existent treatment plants is not well established.^13^ Pakistan is an LMIC in South Asia, where over 1.57 million confirmed cases and more than 30,000 deaths have occurred due to SARS-CoV-2, as of November 28, 2022.^14^ The largest city is Karachi with a population of more than 20 million. The city is divided into 6 administrative units or ‘districts’, of varying population sizes. This project was undertaken in Karachi’s District East that has a population of over 2.8 million. As is common in large cities of LMICs, Karachi has a mix of formal and informal settlements owing to rapid and unplanned urbanization. The sewage system has also followed suite, with many informal drains and channels opening into two main conduits or ‘nallahs’ that empty directly into the sea. Here, we present the feasibility and usability/utility of wastewater surveillance in one such setting in Karachi, Pakistan where untreated wastewater samples were collected from an open sewage channel (*nallah*) over a period of 8 months.

## Methods

### Study site and sample site selection

The health office of District East maintains a database of cases, reported by laboratories within its perimeters for the province-wide COVID-19 surveillance program. In the absence of accurate maps of the district’s sewage network, we selected sampling sites for the study by using 10-m resolution digital satellite images or ‘blue-line maps’ of the District East. The maps (available through www.es.world), provide imagery of the possible flow of sewage along the topographical gradient^15^ (Supplementary Figure S1A and B). Sampling sites were selected after considering factors such as accessibility, minimal industrial waste contamination and overlap of the sewage catchment area with adjoining districts, flow of the stream, and a catchment area population size or ‘sewershed’ of greater than 50,000. Initially, two sites were selected; a third site was added on November 21, 2021, and a fourth site on December 4, 2021 (Supplementary Figure S1C). The combined population of the catchment area of all four sites was over one million.

### Wastewater sample collection

We collected 6 litres of raw sewage twice a week from each site as described above. Collection and filtration were done on-site using all aseptic precautions; the sample collection team wore personal protective equipment and were trained in disinfection and safe handling of equipment. We used Bag Mediated Filtration System (BMFS) technique to collect six litres of wastewater twice weekly from each site.^9^ The BMFS bag is a thick, water-proof bag that is open at the top. A rope is tied firmly at upper end of the bag and the bag is lowered into the stream against the current to collect 6 litres of wastewater. After collection, the bag is fixed upright on a tripod stand and 15–20 min are allowed for sediments to settle down. A ViroCap filter is then attached with a tube to the lower end of the bag and water is allowed to flow into it under gravitational influence. As the water passes through the filter, microorganisms such as viruses are adsorbed through electrostatic interactions.^16^

All equipment, including the ViroCap filter, were carefully disinfected externally and packed separately in biohazard bags for transportation to the laboratory for disposal or disinfection, as relevant. The filter was labelled with the laboratory identification number, packed, and sealed in a biohazard bag and placed upright between ice packs in a temperature-maintained cooler (2–8 °C). The sample was transported within a span of 30–40 min to the biosafety level-2 (BSL-2) Infectious Diseases Research Laboratory (IDRL) at the Aga Khan University for immediate processing.

### Laboratory processing: sample concentration and RNA extraction

The primary concentration of wastewater included filtration, followed by elution with 300 ml of Beef extract (1.5% w/v BBL Beef Extract Powder, Becton Dickinson, Franklin Lakes, USA; Part # 212303 Becton Dickinson, Franklin Lakes, USA). Secondary concentration was performed using skimmed milk flocculation and centrifugation at 3500×*g*, 4 °C for 30 min.^17^ The QIAamp Fast DNA Stool MiniKit (QIAGEN; Coleman Company, Inc., USA) and a modified extraction process adapted from Houpt's Laboratory at the University of Virginia were used to isolate both DNA and RNA from pellets obtained during secondary concentration from BMFS samples.^18^ The sample underwent a lysate preparation process and included mechanical disruption (bead-beating) followed by incubation of the suspension for 5 min at 95 °C for the removal of inhibitors, purification, and elution of DNA and RNA using spin columns. Extrinsic controls MS2 were added to each sample during the lysate preparation to evaluate extraction and amplification efficiency. The extracted total nucleic acid (TNA) was eluted in 200 μl of elution buffer and then stored at −70 to −80 °C until PCR testing.

### Viral RNA detection through RT-qPCR

The SARS-CoV-2 Wastewater RT-qPCR Promega kit (Part # CS317402) was used to target the N1 and N2 regions of the nucleocapsid gene, and envelope (E) gene. Pepper Mild Mottle Virus (PMMoV), an RNA virus commonly found in wastewater, and an internal amplification control (IAC) were used as internal process control. An in vitro transcribed positive control RNA and nuclease free water were also used to check the efficacy of the PCR. MS2 was spiked during extraction to monitor the efficiency of the RNA extraction and detected in all samples with Ct <32.

### Statistical analysis

The District East health Office (DHO-East) maintains a database of all reverse transcriptase PCRs (RT-PCR) confirmed COVID-19 cases reported from the labs and testing facilities located within the district. Through mutual collaboration, deidentified epidemiological data during the study period (June 2021–January 2022) were used for comparison with the viral gene copies in the collected sewage samples using the newly developed distributed lag regression model. Supplementary Figure S1A and B compares the trend of viral gene concentrations from samples collected from Sites 1 and 2 with the reported incident cases of COVID-19 in the district during the study period. The same pattern was observed from samples collected from Sites 3 and 4. However, as these sites were included late in the study, data points were too few to allow for meaningful interpretation. Correlation was also assessed between the incident cases in the district and the observed Ct value (cyclical threshold? Threshold cycle) of the samples during the same time-period as shown in Supplementary Figure S3.

SARS-CoV-2 RNA concentrations were quantified in raw wastewater for each collection time point using the following formula:

$$\frac{\left[\left(\frac{Gene\;copy\;per\;\mu l}{Vr}\right)\times Ve\times (\frac{Vf}{Vc})\right]}{Vi}\times 100$$

where Vi is the initial volume of sample concentration in ml; Vf is the final volume of sample after concentration in ml; Vr is the volume of RNA template used per PCR reaction in μl; Ve is the final volume of RNA eluted from RNA extraction in μl; Vc is the volume of sample after concentration in ml.

### Distributed lag regression model

Estimation of the relationship between wastewater RNA concentration and COVID-19 cases was carried out using a distributed lag negative binomial regression model within a hierarchical Bayesian framework, allowing correct quantification of uncertainty in the estimated associations after accounting for the fact that RNA concentrations were not collected on each day (i.e., missing data) and many were below the limit of detection (LOD) i.e., censored.

In Stage 1 of the framework, we introduce a model for the two sewage RNA concentrations (N1, N2) collected on a given day (averaged across District East) that describes their observed values using latent variables which are either censored or directly observed (i.e., below or above the LOD, respectively). These latent variables represent the true, but sometimes unobserved, RNA concentrations and are modelled jointly using random effects that allow for autocorrelation across time. The joint modelling accounts for the likely positive correlation between concentrations measured on the same day while the autoregressive random effects accommodate correlation in the concentrations across time. In combination, the Stage 1 model allows for prediction of the true or latent concentrations on days where data were not collected and/or the collected value(s) were below the LOD.

In Stage 2, the true RNA concentrations, which are either directly observed or predicted/imputed from Stage 1, are used within a negative binomial regression model to explain variability in the daily COVID-19 case/hospitalisation data. Specifically, the average of the two true log-scaled RNA concentrations on a given day are input into the regression model at different daily lag periods ranging from 1 to 14 days prior. The corresponding regression parameters that describe the associations between these lagged concentrations and the outcome counts are assigned an autoregressive Gaussian process prior distribution to account for the fact that parameters from nearby lags are likely to be more similar and to mitigate the impact of including highly correlated predictors into the regression model (e.g., multicollinearity).^19^^,^^20^ We also include a day-of-week indicator variable in the model to account for data reporting variability across the week. The negative binomial regression model naturally accounts for overdispersion that often is present when analysing count data, ensuring more accurate statistical inference.

Stages 1 and 2 are carried out jointly within a hierarchical Bayesian framework so that uncertainty in the prediction/imputation of RNA concentrations is propagated to the case/hospitalization model. To complete the model specification, we opted for weakly informative prior distributions to allow the data to drive the inference rather than our prior beliefs. The model is fitted using the rjags package within R statistical software.^21^ In total, we collected 10,000 samples from the joint posterior distribution after removing the first 10,000 iterations prior to convergence of the model and thinning the remaining 100,000 by a factor of 10 to reduce posterior autocorrelation. Convergence was assessed by visually inspecting individual parameter trace plots and calculating Geweke’s diagnostic for each parameter of interest.^22^ Watanabe–Akaike information criterion (WAIC) was used to compare the full model with one that excluded wastewater RNA concentrations entirely.^23^ Full details on the newly developed method are given in the Supplementary Material.

### Sequencing using iSeq100 and MiSeq

Sequencing was performed using ARTIC-NEB V3 and V4 protocols. Briefly, the RT-PCR-based ARTIC protocol was used to obtain the amplicon from the extracted RNA, followed by FS DNA library preparation kit (New England BioLabs; Ipswich, Massachusetts, USA) to create the amplicon library. Sequencing was performed using iSeq100 (146 × 2 bps of read length) and MiSeq (71 × 2 bps of read length) platforms which generated raw data in FASTQ compressed format. The samples that passed all quality controls i.e., read filtering, adapter trimming, primer removal, were analysed using KALLISTO^24^ along with customised time range specific databases. As the final output of KALLISTO pipeline, prediction files were generated containing the percentage abundance of SARS-CoV-2 lineages identified per sample. Files were merged into a single 2D matrix file in which rows represent samples and columns represent the abundance of lineages. Wastewater variant abundance was compared with clinical samples obtained from Nextstrain Pakistan build.^25^

**Fig. 1:**
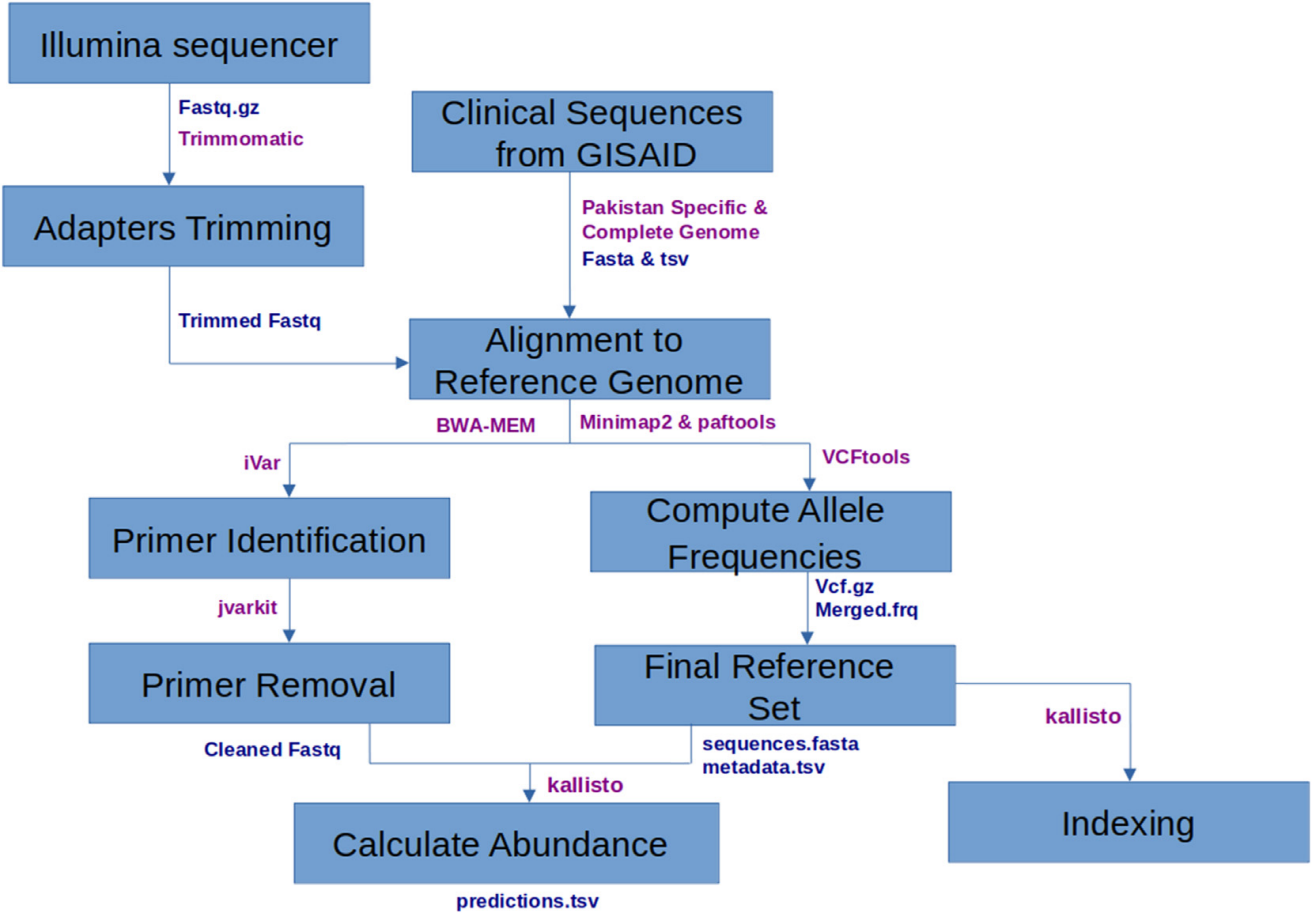
Workflow of bioinformatics analysis: quality control of raw reads followed by calculating variant abundance using reference database built from Pakistani clinical sequences available on Global Initiative on Sharing All Influenza Data (GISAID) database (output formats are coloured as blue, tool names are coloured as purple).

Raw reads generated from iSeq100 and MiSeq were subjected to quality filtering. Adapters were removed using Trimmomatic 0.39,^26^ aligned the trimmed reads to a reference genome (GenBank MN908947.3) with BWA-MEM v0.7.1725,^27^ and subsequently identifying primer sequences using iVar 1.0^28^ and removing them with jvarkit (version 3f7bba5f9)^29^ (Fig. 1). Filtered samples and reference set were used as an input for KALLISTO 0.46.0^24^ to identify distribution of multiple SARS-CoV-2 lineages. PANGO lineages were replaced by WHO labels (Variants of Concern or VOCs and Variant of Interest or VOIs). Samples from each month were pooled to get average abundance of SARS-CoV-2 variants for each month.

### Building genomic reference sets

Reference sets were built by selecting Pakistan-specific and high-quality complete representative of SARS-CoV-2 genomes per lineage from the GISAID [https://www.gisaid.org/] database. Inclusion criteria was to select sequences within the windows of −3 months and +1 month centred at wastewater sample collection month (Table 1). Based on sample collection month, eight reference sets were built to acquire the actual abundance of SARS-CoV-2 variants circulating at that period. A consensus genome of wastewater sequences was generated using web-based CZ ID consensus genome pipeline.^30^ Quality of genomes was assessed using NextClade^31^ and passed sequences were submitted to GISAID.^32^

**Table 1:** Total number of GISAID sequences assigned in each reference set (month-wise).

Sample collection month	Reference set	Number of sequences
June 2021	March–July 2021	823
July 2021	April–August 2021	1126
August 2021	May–September 2021	1063
September 2021	June–October 2021	1027
October 2021	July–November 2021	799
November 2021	August–December 2021	745
December 2021	September 2021–January 2022	495
January 2022	October 2021–February 2022	436

GISAID: Global Initiative on Sharing All Influenza Data database.

### Role of the funding source

Funder had no role in study design, data collection, data analysis, data interpretation, or writing of the report.

## Results

From June 10, 2021 to January 17, 2022, we collected 151 early morning raw, untreated sewage samples, using BMFS technique, from four sampling sites in District East of Karachi. A total of 123 out of 151 (81.5%) sewage samples were positive for the presence of N1, N2, or E gene on reverse transcription polymerase chain reaction (RT-PCR). The concentration of SARS-CoV-2 RNA (log gc/L) was directly proportional and predated the rise and fall of the number of cases in the district (Supplementary Figure S2A and B). We were not able to find much utility of the E-gene and therefore did not use it for further analysis.

**Fig. 2:**
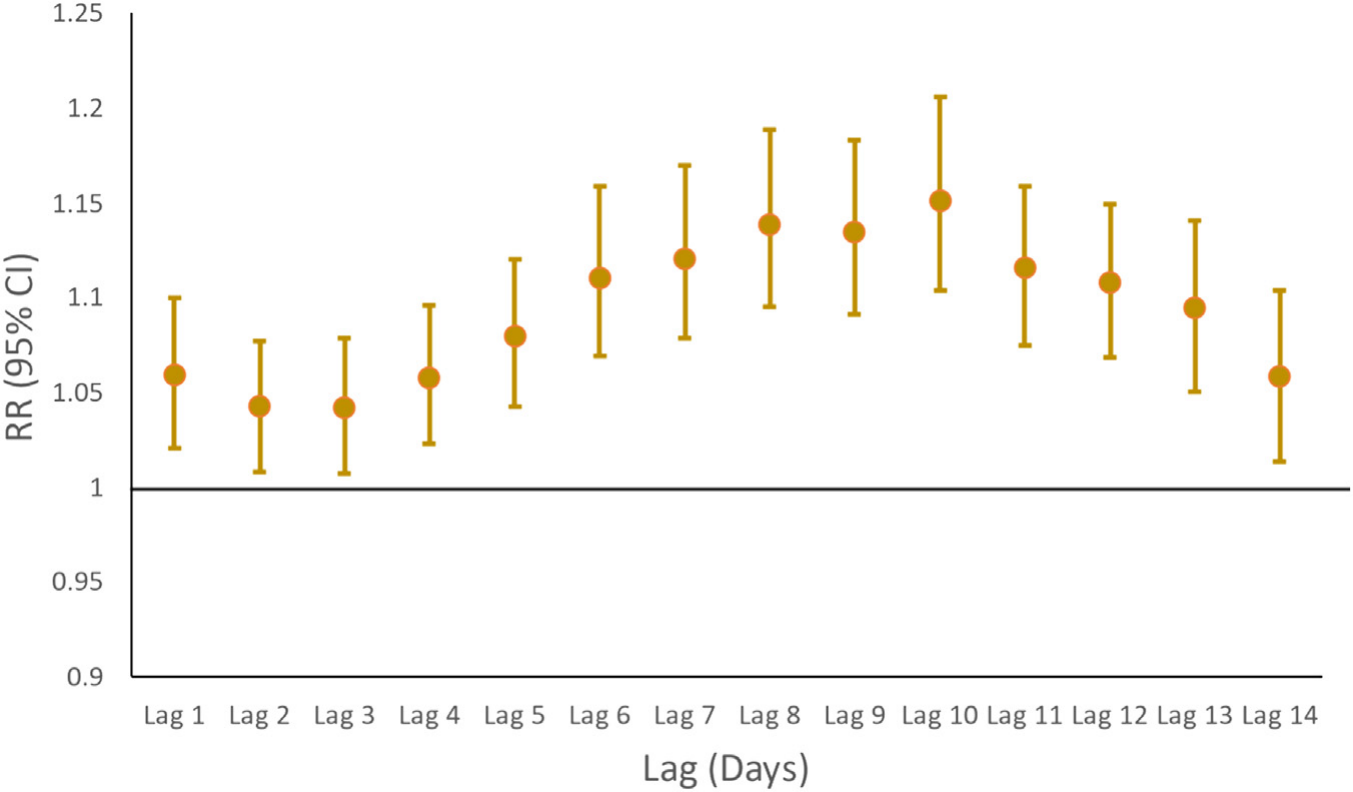
Results from the hierarchical Bayesian distributed lag negative binomial regression model of sewage RNA concentration against the observed COVID-19 cases in District East, Karachi, Pakistan. The plotted coefficients are posterior mean estimates of the risk ratios with their corresponding 95% quantile-based credible intervals.

**Fig. 3:**
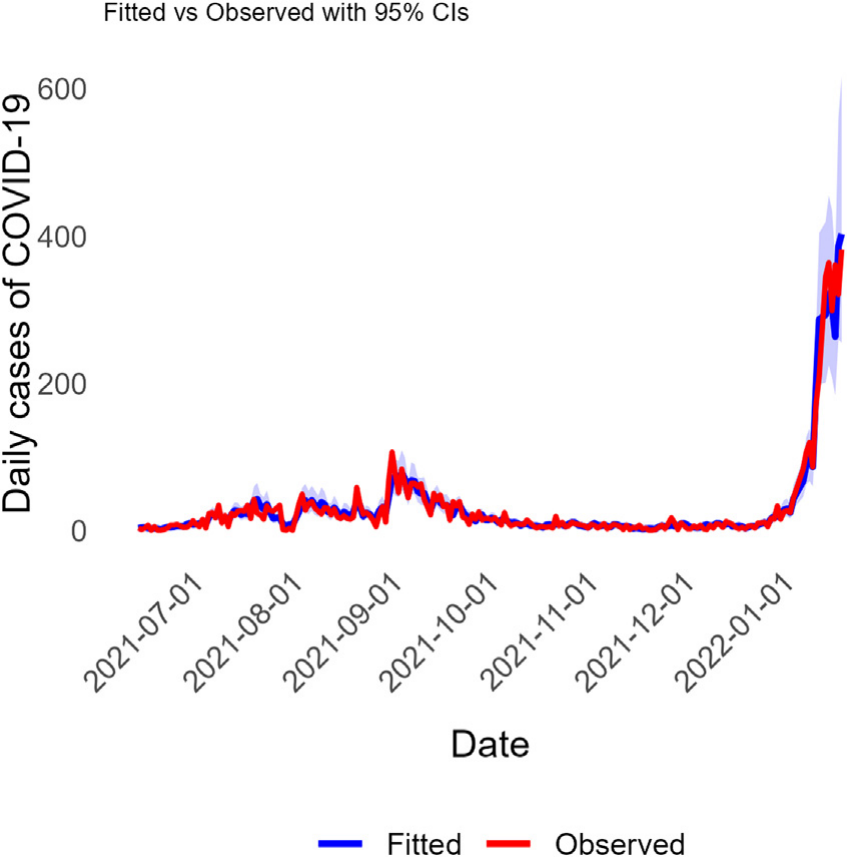
Observed (red) vs. model fitted (blue) case counts for COVID-19 between June 2021 and January 2022. Model fitted estimates of COVID-19 cases in District East from the hierarchical Bayesian distributed lag negative binomial regression model which used sewage RNA concentrations as predictors are plotted against the corresponding observed COVID-19 cases in District East, Karachi, Pakistan.

In Fig. 2, we display estimated risk ratios (posterior means and 95% quantile-based credible intervals or CrIs) from hierarchical Bayesian distributed lag negative binomial regression model of N1 and N2 RNA average (across District East) concentrations (log scale) at a specified number of days prior. CrIs entirely above one suggests that increased RNA concentration during that lag period is associated with an increase in reported cases. Results show that average sewage RNA concentrations at each lag (1–14 days prior) are associated with the respective current days cases, with a peak association observed on lag day 10 (RR: 1.15; 95% CrI: 1.10–1.21). A plot of the model with fitted and observed case counts is shown in Fig. 3 and suggests that sewage RNA concentrations accurately predict the case data. Additionally, WAIC suggested that the full model which included RNA concentrations as predictors was preferred over a simpler version of the model that did not. When the same model was applied to hospitalizations data, the results suggested that sewage RNA concentrations were not predictive of the trends in numbers of hospitalized COVID-19 cases (Supplementary Figure S3).

### Genomic sequencing

**Fig. 4:**
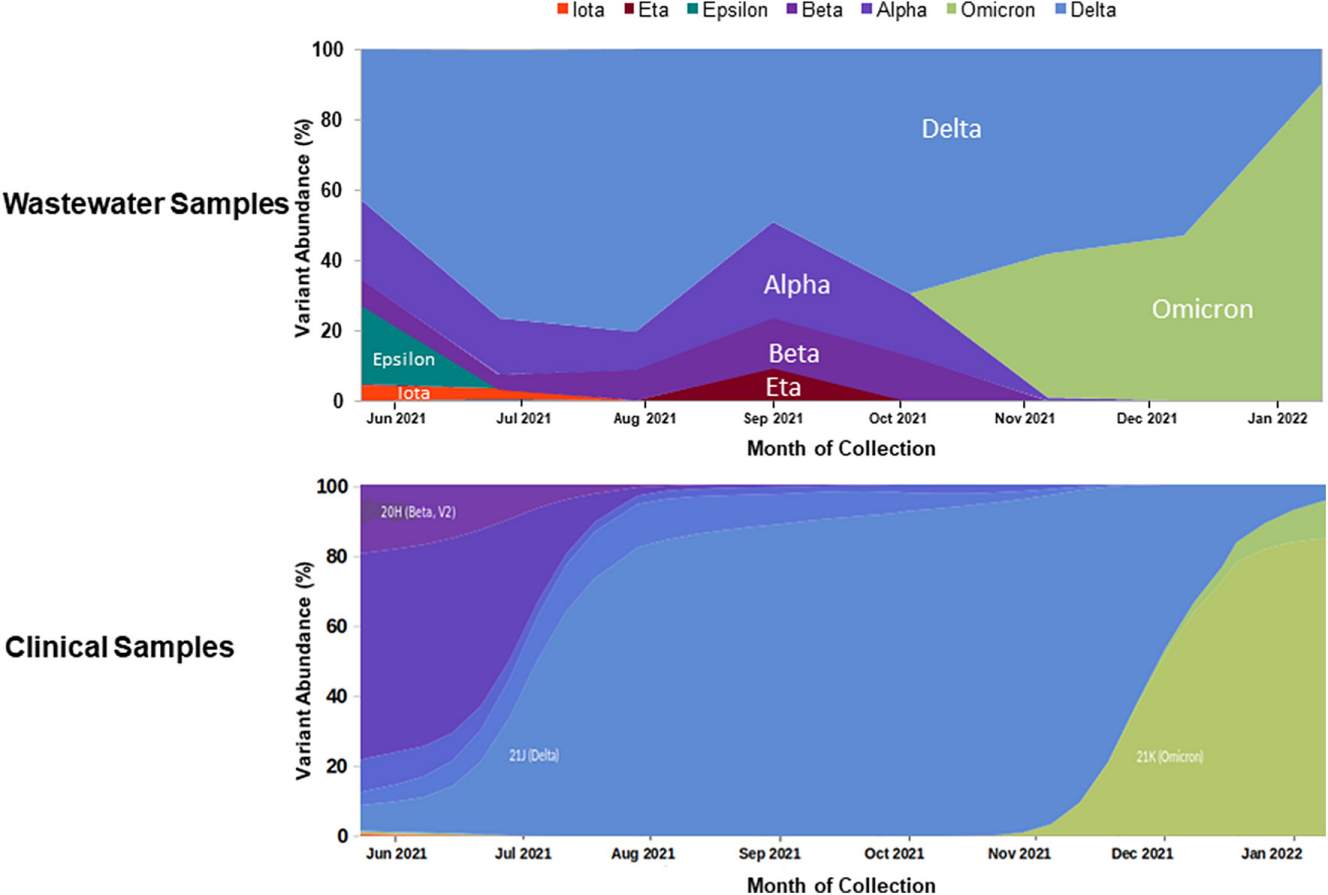
SARS-CoV-2 variants abundance (%) in Pakistani wastewater and clinical samples.

We used Illumina iSeq100 and MiSeq platforms for genomic sequencing 123 positive wastewater samples. This generated 246 pair-end FASTQ files which were analysed using the KALLISTO pipeline that generated a total of 120 prediction files post quality control checks^24^ (Fig. 1). The prediction files contained the percentage abundance of SARS-CoV-2 lineages identified per sample (Table 2). Fig. 4 describes the relative abundance of SARS-CoV-2 variants in the sewage samples compared with the prevalent variants from the clinical samples as available on Global Initiative on Sharing All Influenza Data (GISAID) database for Pakistan (Table 1).^25^ The figure shows that during June–September 2021, other variants could also be seen in the sewage samples although the delta variant was predominant. Of note, the relative abundance of omicron variant became evident 33 days earlier than the first clinically reported case (December 2022).

**Table 2:** SARS CoV-2 variant abundance of 120 wastewater samples (month-wise).

Sample collection month	Alpha (%)	Beta (%)	Delta (%)	Epsilon (%)	Eta (%)	Iota (%)	Omicron (%)
June 2021	23	7	43	23	1	4	0
July 2021	16	4	76	0	0	3	0
August 2021	11	9	81	0	0	0	0
September 2021	27	14	49	0	9	0	0
October 2021	18	13	69	0	0	0	0
November 2021	1	0	58	0	0	0	41
December 2021	0	0	53	0	0	0	47
January 2022	0	0	10	0	0	0	90

## Discussion

We found that SARS CoV-2 RNA concentration in sewage samples could reliably predict incidence of clinical cases in District East of Karachi, Pakistan. Further, next generation sequencing of the samples was able to identify variant abundance as well as emergence of novel variants earlier than sequencing data from clinical samples. To the best of our knowledge, this is the first use-case to demonstrate the utility of direct collection of raw sewage from an ill-defined network for surveillance of COVID-19. This study provides the roadmap for similar settings in Asia and Africa, where sewage networks are poorly laid out and wastewater treatment plants are non-existent.

Our study commenced just before a sharp increase of cases in Karachi when the delta variant dominated the community spread of infection. Our analysis demonstrates a strong correlation between wastewater viral RNA concentration and reported number of clinical cases, with a clear lead time of 14 days. As noted by Hewitt and Olesen,^33^^,^^34^ in the absence of an active clinical surveillance system, there is increased reliance on symptom-based testing, that is, when a case seeks health care after onset of clinical illness.^35^ This leads to an underestimation of the true disease prevalence as most asymptomatic and subclinical infection cases remain unidentified. The same reasons can partly explain the prolonged lead time.^8^^,^^36^^,^^37^ Longer lead times may also reflect delays in reporting of results.^33^ As demonstrated by Peccia and colleagues, lead time may be as little as two days in areas with a robust case surveillance system.^19^ Similar correlation but without appreciable lead times (−2 to +3 days) were reported by Feng and colleagues from Wisconsin.^38^ Our study shows that wastewater-based surveillance can signal an impending outbreak in underserved areas, especially those with weak surveillance systems.^17^^,^^34^

Establishing a waste-water surveillance system in a LMIC setting, such as ours, posed multiple challenges. Our study lays out practical approaches to wastewater surveillance that can be adapted by communities and municipalities with similar informal sewage systems. The first challenge was the absence of a well-defined sewage network due to which accurate estimation of sewage flow and population size of the catchment area could not be reliably ascertained. We circumvented this problem by using 10-m digital elevation model (DEM) maps (www.es.world). These maps infer local sewage network based on the topographical gradient of an area and can help to identify sampling sites with a catchment area of suitable population size. Second challenge was that Karachi does not have functional wastewater treatment plants (WWTPs). An advantage of sampling primary sludge from WWTPs is that it yields a higher concentration of the virus at lower volumes.^16^ On the other hand, when collecting samples from main sewage channels, a large volume is required that must be concentrated, which adds to the complexity and cost. We used BMFS, a type of grab method to collect 6 litres of raw sewage which was filtered on-site before transportation to the laboratory. Despite the large volume and costly equipment, this method concentrates the sample on-site and reduces the effective volume.^16^^,^^39^ It also eliminates the risk of spillage and contamination that could occur during transportation and handling. Once in the laboratory, skim milk flocculation was used to further concentrate the samples which is inexpensive and is unaffected by supply constraints due its wider availability.^16^ We could not opt for 24-h composite sampling due to logistical issues. Composite sampling could have increased the accuracy as it can account for diurnal variation in sewage viral RNA levels.^36^ We addressed this limitation by collecting samples early in the morning as the flow rate and human faecal load is generally higher at the time.^40^ As shown by Liu and colleagues, using Moore swab is another method that is cost-effective and has better sensitivity than grab samples, especially when targeting smaller populations.^41^ However, this method requires that the equipment be placed at the collection point for a longer time-period (preferably 24 h). Further, the LOD may vary with higher turbidity of wastewater. Both factors were prohibitive for us as we collected our samples from an open sewage channel. Additionally, we collected samples biweekly from each site for a more accurate prediction of trend over time. Daily sampling was not feasible due to higher cost. Further, because of the long lab turn-around time from sample collection to analysis, infrequent collection (once a week) would have reduced the information necessary for meaningful inference. Estimating the precise residential location of the cases also posed a problem due to GIS (Global Information System) errors, incomplete or missing information. We included district-wide data of reported cases for statistical modelling as it gave a more accurate depiction of the transmission trends in the community.

Our study had many strengths; for one, this was among the first studies from Pakistan to perform high throughput sequencing (HTS) of SARS-CoV-2 in wastewater samples. A systematic review highlighted the importance of ES as a tool for monitoring spread of VOCs through genomic sequencing of the viral RNA from sewage samples.^42^ Sequencing from wastewater samples is much more complex than clinical samples due to low concentration of viral RNA, presence of multiple variants in the same sample, and contaminants.^8^^,^^38^^,^^43^ Moreover, the bioinformatics pipelines to analyse wastewater sequencing data are not well-established. Nonetheless, wastewater sampling can provide valuable population-level variant information on SARS-CoV-2, including important data on variant distribution that could be missed in clinically derived sequencing data. In the latter, genomes are sequenced predominantly from those symptomatic cases from which genetic material can be derived. Hence, the sequenced samples represent just a small percentage of those shedding virus in a community and present only the more dominant or virulent strain. On the other hand, sequencing wastewater samples provide a holistic picture of the variants circulating in the population. This was evident from the variant abundance in wastewater samples throughout the study duration, while there was a preponderance of the more dominant variants in the clinical samples, such as the delta variant between July and September and the omicron variant after November 2021.

Our study strengthens the existing evidence regarding the immense potential of wastewater surveillance as a public health measure for indicating infection outbreaks and detecting emerging variants ahead of time. This can have potential therapeutic and interventional implications. Newer variants, such as the omicron and its various strains, are more infectious because of their enhanced ability to evade the immune system.^44^ This led to reduction in effectiveness of the vaccines that were developed against earlier strains and prompted the use of booster doses and variant specific alterations of the vaccines. On therapeutic front, the previously approved treatment modalities continue to be the mainstay of management of moderate to severe illness caused by the newer variants. It remains to be seen how much this may change if the virus develops significant mutations and gains resistance against the more commonly used drugs and therapies.^44^

Wastewater-based surveillance and genomic level analyses are useful for monitoring and predicting temporal trend of SARS-CoV-2 at the community-level. Such a system can be set-up even in low resource settings to provide early warning so that appropriate measures can be taken to mitigate the risk of transmission to a larger population.

## Contributors

These authors contributed equally: NA, MIN, FK, and WK. These authors jointly supervised the work: MIN, FJ, SBO, NA, MIN, WK, FK, AAM, and JLW drafted the initial manuscript. SBO, FJ, UM, AI, WT, IY, JSM, AMK, AM, SMA, FA, SK, MMA, and AI critically read and contributed to the manuscript. FKd, JLW, AAM, and HK contributed to the figures and graphs. FK and FA provided data from the wet lab; WK and SK provided the genomic sequencing figures.

## Data sharing statement

All data supporting the findings of this study are available within the article and can also be viewed on WSphere (https://sphere.waterpathogens.org/dataset/62d5deda-7d7b-483c-b4da-1eb80882df2c). Genomic data are available on GISAID (www.gisaid.org) or NCBI (BioProject ID: PRJNA860755). The data related to the study and codes used for statistical data analysis are available from corresponding author upon reasonable request without any restrictions.

## Editor note

The Lancet Group takes a neutral position with respect to territorial claims in published maps and institutional affiliations.

## Declaration of interests

This study was funded by PATH (grant numbers: GAT.583722-01707644-CRT and GIF.583820-01712491-CRT), the Bill & Melinda Gates Foundation (grant number INV-021602) and by the Global Innovation Fund (PATH Covid-19 Environmental Surveillance, grant number 583820) provided to MIN. All authors agree there are no other conflicts of interest. The findings and conclusions contained within are those of the authors and do not necessarily reflect positions or policies of the funders.
