# Supplementary material for: Environmental surveillance for COVID-19 using SARS-CoV-2 RNA concentration in wastewater – a study in District East, Karachi, Pakistan
Source: Lancet Reg Health Southeast Asia. 2023 Oct 23;20:100299. doi: 10.1016/j.lansea.2023.100299 (PMC10794106; doi:10.1016/j.lansea.2023.100299)
Supplement: Supplementary Material [file mmc3.docx]

**Supplementary information**

**Environmental surveillance for COVID-19 using SARS-CoV-2 RNA concentration in wastewater - a study in District East, Karachi, Pakistan**

*Nadia Ansari^1#^, Furqan Kabir^1#^, Waqasuddin Khan^2#^, Farah Khalid^1^, Amyn Abdul Malik^3,4^, Joshua L. Warren^5^, Usma Mehmood,^1^ Abdul Momin Kazi^1^, Inci Yildirim^3,4,5^, Windy Tanner^5^, Hussain Kalimuddin^1^, Samiah Kanwar^1,2^, Fatima Aziz^1^, Arslan Memon^6^, Muhammad Masroor Alam^7^, Aamer Ikram^8^, John Scott Meschke^9^, Fyezah Jehan, ^1,2^, Saad B. Omer,^3,4,5,10^, and Muhammad Imran Nisar^1,2#*^*

^1^Department of Paediatrics and Child Health, Faculty of Health Sciences, Medical College, The Aga Khan University, Stadium Road, Karachi-74800, Pakistan.

^2^CITRIC Centre for Bioinformatics and Computational Biology, Department of Paediatrics and Child Health, Faculty of Health Sciences, Medical College, The Aga Khan University, Stadium Road, Karachi-74800, Pakistan.

^3^Yale Institute for Global Health, Yale University, New Haven, CT, USA

^4^Section of Infectious Diseases and Global Health. Department of Paediatrics, Yale School of Medicine, Yale University, New Haven, CT, USA

^5^Yale School of Public Health, Yale University, New Haven, CT, USA

^6^District Health Office (East), Karachi, Pakistan

^7^World Health Organization: Islamabad, Chak Shahzad, Islamabad, Pakistan

^8^National Institutes of Health, Chak Shahzad, Islamabad, Pakistan

^9^University of Washington, Seattle, Washington, USA

^10^Yale School of Nursing, Orange, CT, USA

^#^Contributed equally

*Corresponding Author

Department of Paediatrics and Child Health, Faculty of Health Sciences, Medical College, The Aga Khan University, Stadium Road, Karachi-74800, Pakistan.

*E-mail address:* [imran.nisar@aku.edu](mailto:imran.nisar@aku.edu) (M.I. Nisar)

Table of Contents

**Distributed lag regression model**2

Statistical analysis2

Alternative Multivariate Normal Specification3

**Supplementary figures5**

Supplementary Figure 1A: Map of Karachi showing District East5

Supplementary Figure 1B*: Blue-line mapping …6

Supplementary Figure 1C: Study sampling sites .………………………………………………………………………………………………… 6

Supplementary Figure 2A: Correlation of COVID-19 incident cases with viral gene concentration from Site 1 samples……………………………………………………………………………………………………………………………………….….………….……. 7

Supplementary Figure 2B: Correlation of COVID-19 incident cases with viral gene concentration from Site 2 samples …………………………………………………………………………………………………………………………………………………. 8

Supplementary Figure 3: Hierarchical Bayesian distributed lag negative binomial regression model of sewage RNA concentration against COVID-19 hospitalizations at a tertiary care hospital in District East…………………………… 9

**Supplementary Information**

**Distributed lag regression model**

**Baseline epidemiological data.** Daily COVID-19 case counts, and hospitals admissions from the catchment area were compiled from the database available from the District Health Office of District East. Deidentified data was used for the analysis.

**Statistical analysis***.* SARS-CoV-2 RNA concentrations was quantified in raw wastewater. Estimation of the relationship between wastewater RNA concentration and COVID-19 cases was carried out using a Bayesian framework, allowing us to correctly quantify uncertainty in the estimated associations. For estimating lead times, a distributed lag analysis framework was used. In these analyses, we assumed that the observed sewage testing data represents unbiased estimates of an underlying, unobserved trajectory of viral concentration in the wastewater. We evaluated the association between the underlying trajectory of viral concentration in the sewage at multiple lagged periods and the number of cases/hospitalisations using a distributed lag negative binomial regression model including a random effect to account for overdispersion and autocorrelation in the outcome. The detailed statistical framework is listed below:

**Covid Case Counts Model:**

$$Y_{it}\sim Negative Binomial\left( r,p_{t} \right),$$

$$p_{t}=\frac{r}{r+\lambda_{t}}, \ln\left( \lambda_{t} \right)=\beta_{0}+\mathbf{x}_{t}^{T}\boldsymbol{\gamma}+\sum_{l=1}^{d} g\left\{ \ln\left( z_{1,t-l}^{*} \right),ln\left( z_{2,t-l}^{*} \right) \right\}\theta_{l}$$

- $Y_{t}$: Covid case count on day t
- Negative binomial distribution (as specified):
  - Expected value: $\lambda_{t}$
  - Variance: $\lambda_{t}\left( 1+\frac{\lambda_{t}}{r} \right)$
- $r$: Dispersion parameter, $>0$; small values indicate more overdispersion
- $p_{t}$: Probability parameter that helps define the magnitude of the case counts
- $\beta_{0}$: Intercept parameter
- $\mathbf{x}_{t}$: Vector of covariates; could be empty (does not include an intercept)
  - $\boldsymbol{\gamma}$ the corresponding vector of regression parameters
- $z_{jt}^{*}$: True concentration of virus at target site j (sometimes observed directly, sometimes censored by limit of detection (LOD))
  - $\theta_{l}$: Distributed lag regression parameters
  - $d$: Maximum number of daily lags considered
  - $g(.,.)$: Function of the wastewater concentrations collected at the two different virus target sites (e.g., mean, maximum)

**Wastewater Concentrations Model:**

$$\left\{ \delta_{jt},z_{jt} \right\}$$

- $\delta_{jt}$: Censoring indicator equal to 1 if $z_{jt}^{*}<LOD$ (censored) and equal to 0 if $z_{jt}^{*}\geq LOD$ (not censored)
- $z_{jt}$: Observed wastewater concentration; $z_{jt}=\max\left\{ z_{jt}^{*},LOD \right\}$

$$\left[ \begin{matrix} \ln\left( z_{1t}^{*} \right) \\ \ln\left( z_{2t}^{*} \right) \end{matrix} \right]\sim MVN\left( \left[ \begin{matrix} \mu_{1}+\eta_{1t} \\ \mu_{2}+\eta_{2t} \end{matrix} \right],\Sigma\right)$$

- $MVN(.,.)$: Multivariate normal distribution
- $\mu_{j}$: Virus target site-specific intercept parameters
- $\eta_{jt}$: Virus target site-specific time-series correlated parameters
- $\Sigma$: Two-by-two covariance matrix that describes covariance/correlation between latent concentrations from the different virus target sites on the same day

**Prior Distributions:**

- $r\sim Gamma\left( 0.01, 0.01 \right)$
- $\beta_{0},\gamma_{k},\mu_{j}\sim N\left( 0, {100}^{2} \right)$
- $\theta_{1}\left| \tau_{\theta}^{2}\sim N\left( 0, \frac{\tau_{\theta}^{2}}{1-\rho_{\theta}^{2}} \right); \theta_{l} \right|\theta_{l-1},\rho_{\theta},\tau_{\theta}^{2}\sim N\left( \rho_{\theta}\theta_{l-1}, \tau_{\theta}^{2} \right)$ for $l\geq2$
  - $\tau_{\theta}\sim Uniform\left( 0.00, 100.00 \right)$
  - $\rho_{\theta}\sim Uniform\left( 0.00, 1.00 \right)$
  - Gaussian process connection
- $\eta_{j1}\left| \tau_{\eta}^{2}\sim N\left( 0, \frac{\tau_{\eta}^{2}}{1-\rho_{\eta}^{2}} \right); \eta_{jt} \right|\eta_{j,t-1},\rho_{\eta},\tau_{\eta}^{2}\sim N\left( \rho_{\eta}\eta_{j,t-1}, \tau_{\eta}^{2} \right)$ for $t\geq2$
  - $\tau_{\eta}\sim Uniform\left( 0.00, 100.00 \right)$
  - $\rho_{\eta}\sim Uniform\left( 0.00, 1.00 \right)$
  - Shared $\tau_{\eta}$ and $\rho_{\eta}$ across both virus target sites, but could be separate if necessary
  - Gaussian process connection
- $\Sigma^{-1}\sim Wishart\left( 3, I_{2} \right)$

**Alternative Multivariate Normal Specification:**

- As JAGS (the statistical package) cannot handle vector responses when some entries are missing and others are not
- Instead, use the following conditional version of the distributions to define the full joint distribution of interest

$$\Sigma=\left( \begin{matrix} \omega_{1}^{2} & \rho\omega_{1}\omega_{2} \\ \rho\omega_{1}\omega_{2} & \omega_{2}^{2} \end{matrix} \right),$$

$$\ln\left( z_{1t}^{*} \right)\sim N\left( \mu_{1}+\eta_{1t},\omega_{1}^{2} \right),$$

$$\ln\left( z_{2t}^{*} \right)|\ln\left( z_{1t}^{*} \right)\sim N\left( \mu_{2}+\eta_{2t}+\kappa\frac{\omega_{2}}{\omega_{1}}\left\{ \ln\left( z_{1t}^{*} \right)-\mu_{1}-\eta_{1t} \right\},\left( 1-\kappa^{2} \right)\omega_{2}^{2} \right)$$

- $\omega_{1}\sim Uniform\left( 0.00, 100.00 \right)$
- $\omega_{2}\sim Uniform\left( 0.00, 100.00 \right)$
- $\kappa\sim Uniform(-1.00,1.00)$

The distributed lag model accounts for missing data and limit of detection of the PCR assay during analysis. The model was not stratified by site as there might be a possible misclassification of the daily cases being associated with any given catchment area for the 4 sites. We built the model at the district level. In the article, Figures 2 and 3 show that sewage RNA concentration after adjustment for time lags (0 to 14-day lags) tracked the rise and fall in the daily reported COVID-19 case counts in District East. The model shows that relative to the daily reported case counts, the sewage RNA concentrations were 5 to 13 days ahead. The model does not predict daily hospitalization count very well (Supplementary Data Figure 3). This is probably because a lot of noise in the hospitalization data. The tertiary care hospital in the district is a regional referral center and receives COVID-19 cases not only from the remaining 6 districts in Karachi but also from outside the city.

**Supplementary figures**

**Supplementary Figure 1A:** Map of Karachi showing District East

**
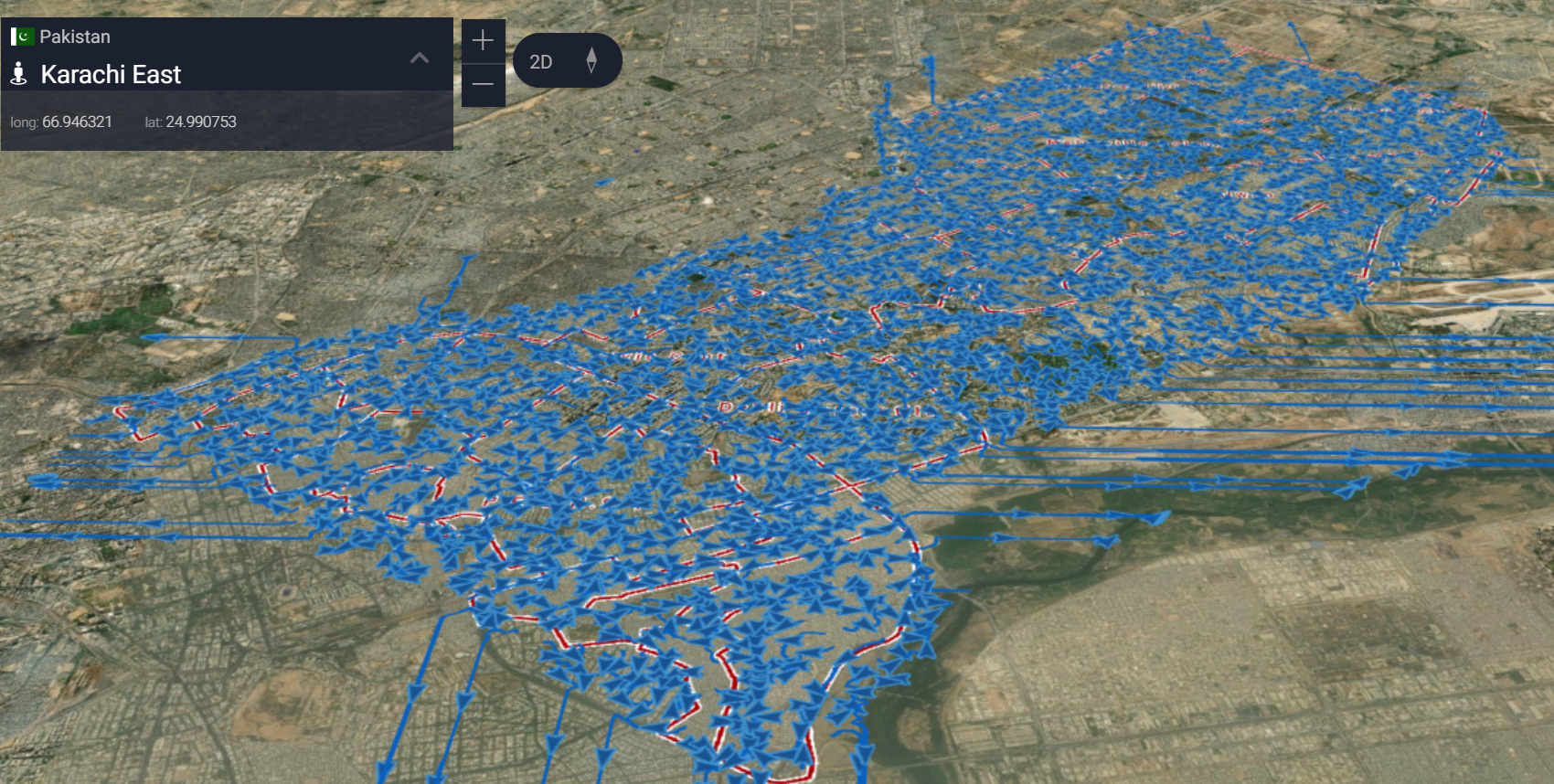
**

Supplementary Data Figure 1B*: Blue-line mapping showing sewage flow based on the topographical gradient

**
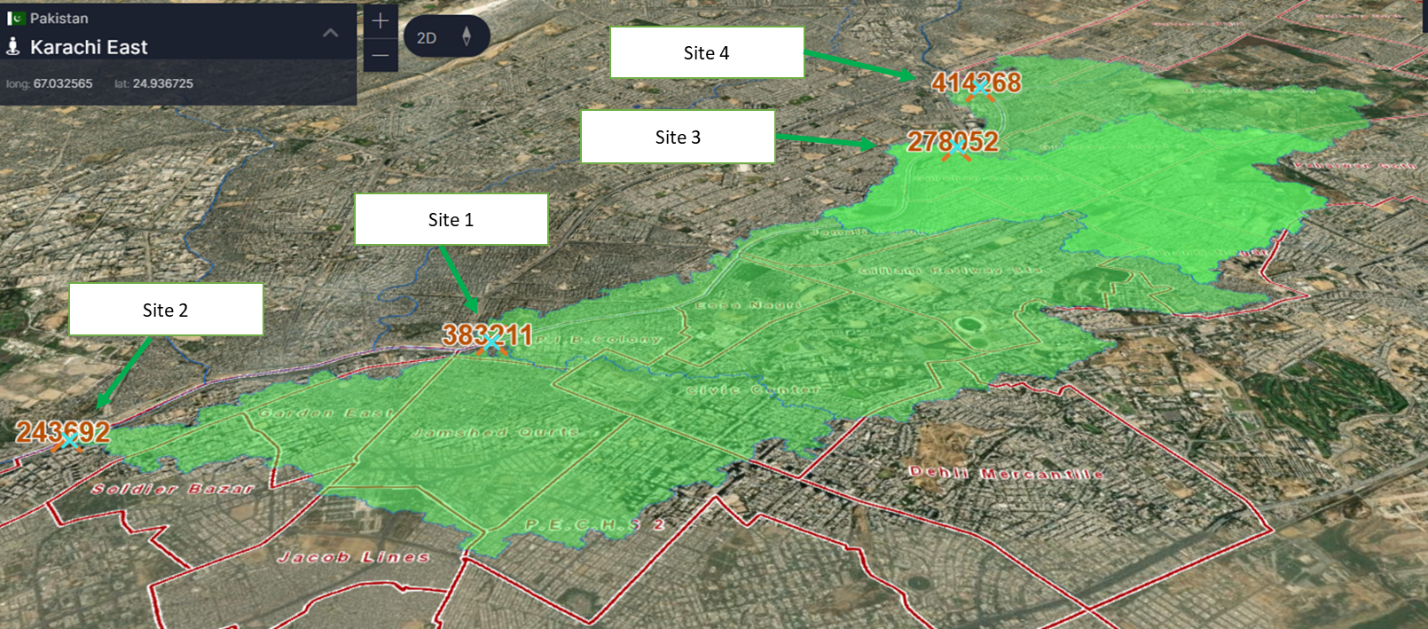
**

**Supplementary Data Figure 1C*:** Satellite map of District East, Karachi, showing the four sampling sites for the study and the approximate population size of the catchment area (sewershed).

*****Source: [www.es.world](http://www.es.world)


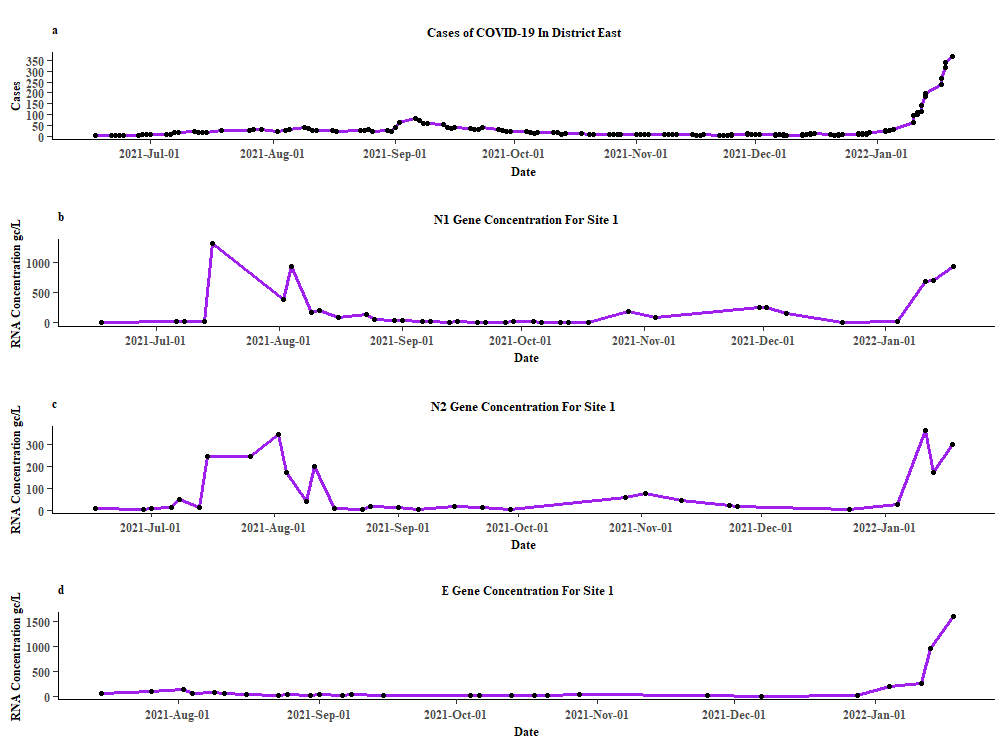


**Supplementary data Figure 2 A:** Panel graph showing the correlation between incident cases in District East (a) with the concentration of SARS-CoV-2 N1(b), and N2 (c) in samples collected from Site 1 from June 2021-January 2022.


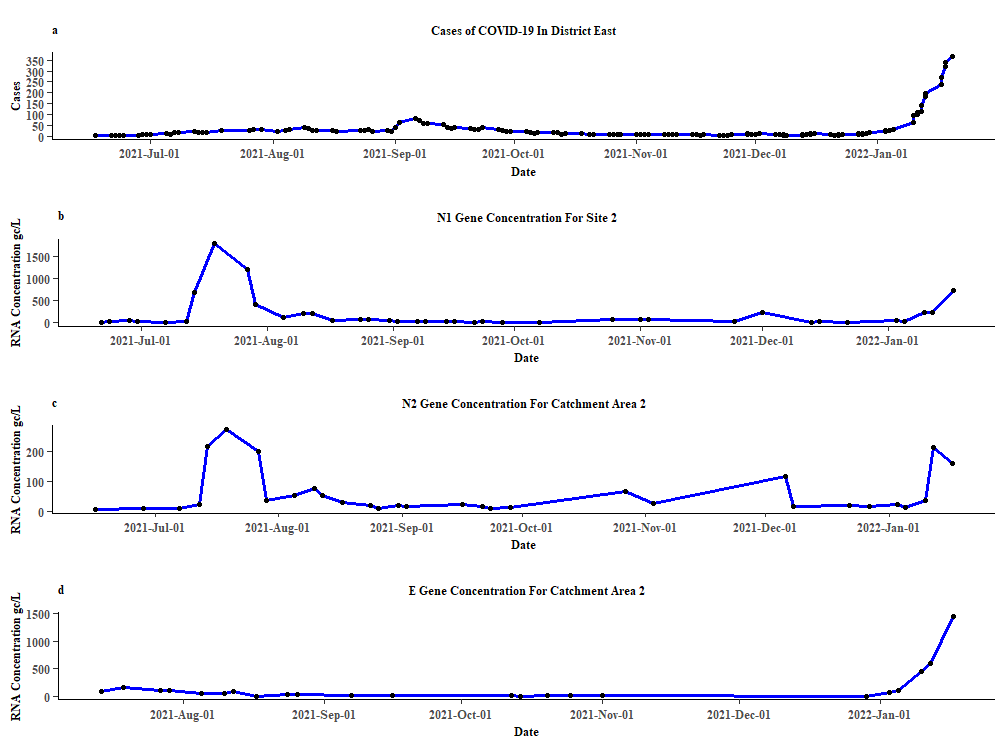


**Supplementary data Figure 2B:** Panel graph showing the correlation between incident cases in District East (a) with the concentration of SARS-CoV-2 N1(b), and N2 (c) in samples collected from Site 2 from June 2021-January 2022.


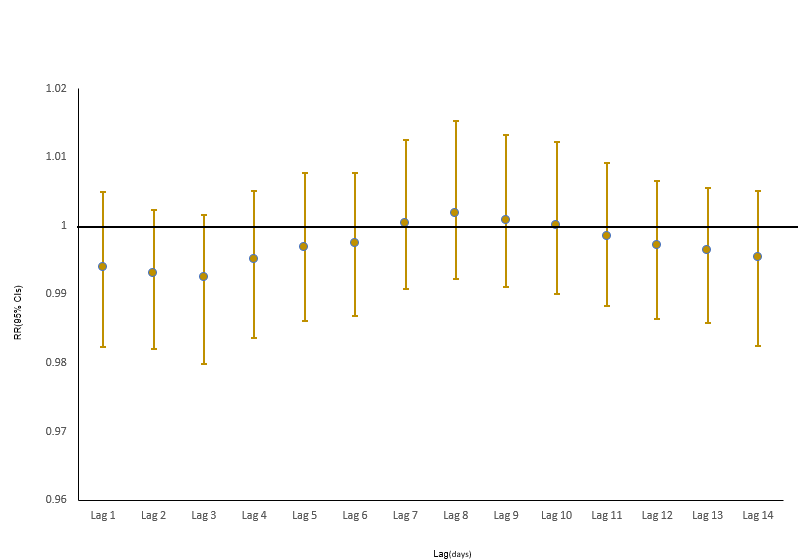


**Supplementary Data Figure 3:** Results from the hierarchical Bayesian distributed lag negative binomial regression model of sewage RNA concentration against the COVID-19 hospitalizations at a tertiary care hospital in District East. The plotted coefficient are posterior mean estimates of the risk ratios with their corresponding 95% quantile-based credible intervals.
